# Effects of a Cloth Panel Containing a Specific Ore Powder on Patients with *Chamaecyparis obtusa* (Cypress) Pollen Allergy

**DOI:** 10.1155/2021/3924393

**Published:** 2021-11-10

**Authors:** Suni Lee, Yukiyoshi Hyo, Shoko Yamamoto, Hiroshi Okamoto, Yoshio Fujii, Hirotaka Hara, Takemi Otsuki

**Affiliations:** ^1^Department of Hygiene, Kawasaki Medical School, Kurashiki-City, Okayama Prefecture, Japan; ^2^Department of Otolaryngology, Kawasaki Medical School, Kurashiki-City, Okayama Prefecture, Japan; ^3^Cosmic Garden Co., Ltd., Kita-ku, Okayama City, Okayama Prefecture, Japan

## Abstract

Pollen allergy to Japanese cedar and cypress is a serious illness that impairs daily life and sleep, especially during pollen season. We have reported that placing a cloth panel containing a specific natural ore powder (CCSNOP) in a room may alleviate the symptoms of hay fever and may also benefit the immune system. This ore is from the Aso mountain range, a volcano on Kyushu Island in the southwestern part of Japan. The purpose of this study was to verify the effect of CCSNOP on cypress pollen. Thirty-one double-blind tests, which investigated cedar pollen allergies, were conducted from February to March 2018 and have already been reported. After this, in early April, 10 of these cases were recruited and all had CCSNOP installed in their bedrooms. Before that, various symptoms and changes in medication were recorded in a “Symptom Diary” and included a mood survey by a questionnaire, stress test using saliva amylase, changes in cypress-specific immunoglobulins IgE and IgG4 by blood sampling, and eosinophil changes. In addition, changes in 29 types of cytokines were investigated. Exposure to CCSNOP relieved symptoms and subjects decreased their intake of medication. There was no change in mood or stress, but eosinophil levels tended to decrease. Although there were no statistical changes in cypress-specific IgE or IgG4, an increase in the former and a decrease in the latter were observed in some individuals during the period of pollen dispersal. Furthermore, levels of GM-CSF and IL8 decreased significantly after use of CCSNOP. The CCSNOP was shown to be effective against cypress pollen allergy, and future investigations will be necessary to observe the long-term effects of CCSNOP.

## 1. Introduction

A condition that causes allergic rhinitis or allergic conjunctivitis due to pollen is called hay fever. Especially in the last two decades, the number of cases has increased remarkably in developed countries [1–3]. Plants that cause pollen allergies include cedar [4, 5], cypress [6], dactylis [7], timothy [8], ragweed [9], and birch [10]. Of course, many more have been reported, but the central ones are cedar and cypress. These are *Chamaecyparis*, *Cryptomeria japonica* (cedar), and *Chamaecyparis obtusa* (cypress) [4–6]. Even in Japan, many cases of pollen allergy include symptoms of rhinitis or conjunctivitis during the pollen dispersal period [11, 12]. Symptoms of rhinitis include sneezing, runny nose, and stuffy nose [11, 12]. Eye symptoms comprise redness, watery eyes, and itch [13]. In Japan, Japanese cedar pollen is often dispersed from late February to the end of March. After that, cypress pollen dispersal continues for about one month [14]. Cypress pollen often occurs mainly in western Japan, and it has been reported that there are many cases of pollen allergy caused by cypress pollen in western Japan [15].

Despite these symptoms, the symptoms usually subside after pollen dispersal has ceased. In a sense, it is a time-limited illness. Cases with symptoms will be accompanied by many adverse effects such as restrictions on daily life, decreased production activity, and lack of sleep, which represent a problem in terms of quality of life [14–16].

As for treatment, as mentioned above, it is not a conventional disease, and so antihistamines are mainly employed as a form of symptomatic treatment. Allergen immunotherapy may also be attempted, depending on the degree of adverse effects on daily life [17, 18]. However, in reality, the reason why allergen immunotherapy has not been more generally employed is that the degree of pollen dispersal varies depending on the year, and cases become asymptomatic after a certain period of time.

For pollen allergies, suitable indoor environments and other alternative therapies may be more beneficial *in lieu* of drugs [19, 20]. Given this possibility, we have been studying the effect of cloth mixed with a special ore powder [21, 22]. Cosmic Garden, a housing manufacturer in Okayama City, Okayama Prefecture, located in western Japan, has been using this ore powder for interior materials for more than a decade. This ore was described in past reports [21, 22]. This ore is from the Aso mountain range, a volcano in Kyushu, an island in western Japan.

Many people utilizing Cosmic Garden materials in their indoor environments have commented that their sleep status has improved and that their pollen allergies are improved. The office of Cosmic Garden is also equipped with similar interior materials, and many customers have remarked that upon entering this office space they experience improvement of their pollen allergy symptoms [21, 22].

Therefore, we tried to verify the beneficial effects of this cloth containing a specific natural ore powder (CCSNOP) at the time of pollen dispersal. Initially, a panel containing CCSNOP was installed, and a one-hour stay experiment was conducted using 20 volunteers prone to pollen allergy [21]. Results indicated an improvement of symptoms and mood of the volunteers. However, regarding the dynamics of eosinophils and changes in cytokines, although there were some items that showed significant differences between the control and CCSNOP panel groups, it was not possible to fully explain these differences.

Therefore, in the next experiment, we conducted an experiment in which volunteers with pollen allergies were asked to install the CCSNOP panel in their bedroom for 2 weeks during the 2018 cedar pollen dispersal period [22]. The results of this study, which we have already reported, showed improvement of symptoms in volunteers utilizing CCSNOP compared to button tiers with control panels within 2 weeks of utilization [22]. Additionally, a significant decrease in use of medication was observed during that 2-week period. The data collected comprised symptom scores, the absolute number of various cells in the leukocyte fraction, the immunoglobulin value, the specific immunoglobulin (Ig) *E* value, and the values of the 29 cytokines before and after panels were installed. When mixed and multiple regression analysis was performed, a formula was derived, which made it possible to determine with significant difference whether the volunteer had been exposed to CCSNOP or control panels. In the formula, levels of granulocyte-macrophage growth stimulating factor (GM-CSF), interleukin- (IL-) 12p40, IgG4 (nonspecific, but general), and eosinophil numbers were extracted as components. This suggests that CCSNOP panels act on the immune system as well as improving the symptoms of pollen allergy [22].

For this second project, the aim was to evaluate CCSNOP against allergic symptoms caused by cypress pollen. It was assumed that the dispersal periods of Japanese cedar pollen and Japanese cypress pollen differ slightly [4–6]. The former occurs from the end of February to the latter half of March [4, 5], while the latter occurs from the end of March to the end of April [6]. Additionally, for Japanese cypress allergy, we had a chance to measure IgE for cypress as well as IgG4 with the collaboration of Siemens Healthcare Diagnostics K.K., Tokyo, Japan [23]. These immunoglobulins were not measured in previous studies. Thus, 10 volunteers were recruited for the aforementioned second project. Although, for the second project, volunteers were living around Okayama (western Japan, just between Hiroshima and Osaka) and Kobe (near Osaka), for this new project, we selected volunteers who were living around Okayama since our laboratory is located in Okayama Prefecture. This time, all 10 volunteers were exposed to CCSNOP panels. Since the sample number was limited, it was difficult to make a comparison with control panels. Then, as before, all 10 volunteers planned to take a survey of mood and stress and blood sampling on January 13 (period with no pollen dispersal), February 24 (just before panel installation for Japanese cedar investigation), March 11 (just after cedar investigation), and June 9 (period with no pollen dispersal) of 2018. In addition to this data, 10 volunteers were exposed to CCSNOP panels for 2 weeks at the beginning of April. During the period comprising 1w before up until 1w after panel installation, volunteers kept a “Symptom Diary.”

In this report, we show results of this third investigation of the “effects of CCSNOP on patients with Japanese cypress pollen allergy” and found that symptoms had improved and that subjects decreased their intake of medication. Interestingly, the number of eosinophils in peripheral blood seemed to decrease. GM-CSF and IL8 levels were significantly lower after use of CCSNOP. Given the results of the second investigation, CCSNOP may act to reduce allergy symptoms in people during the pollen dispersal period.

## 2. Subjects and Methods

### 2.1. Subjects

**Figure 1:**
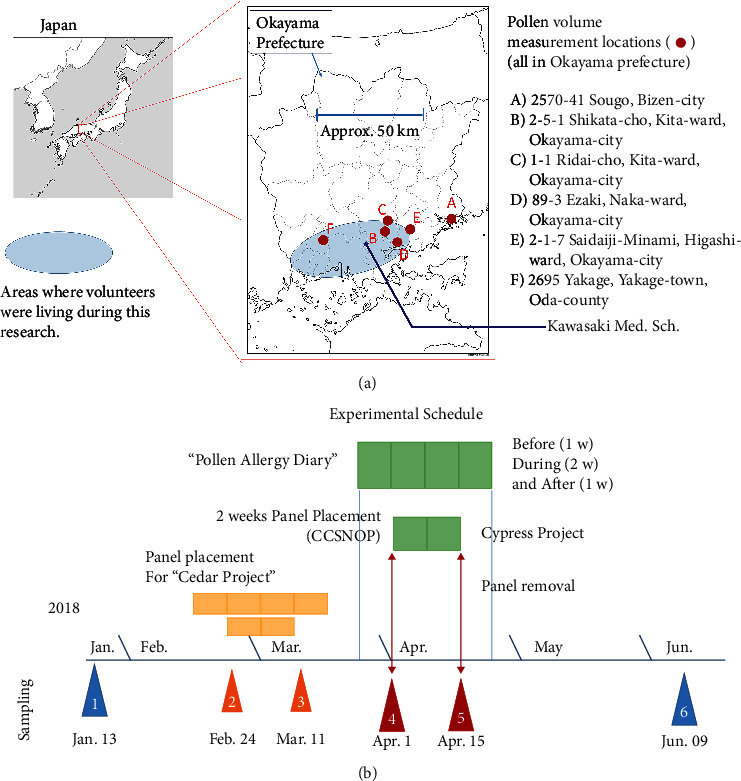
Areas where volunteers resided, measuring spots of cypress pollen (according to the literature shown in Figure 2), and experimental schedule. (a) All subjects were residing in the southern part of Okayama Prefecture, Japan. Okayama Prefecture is located in the western part of Japan. A determination of the amount of cypress pollen at the dates when subjects were exposed to CCSNOP was obtained by inspecting the literature referred to in Figure 3. From this literature, A to F spots represent areas where cypress pollen volume was measured. The location (addresses) of the spots is shown. All spots except A overlapped with areas where subjects were residing. (b) The experimental schedule is shown. This study was based on a previous investigation that examined the effects of CCSNOP on 31 patients with Japanese cedar pollen allergies (“Cedar Project”). The results have been previously published. Ten subjects were then selected from the aforementioned 31 patients, all of whom were residing in the southern part of Okayama Prefecture, as shown in panel (a). For the “Cedar Project,” all subjects were exposed to CCSNOP in their bedrooms from February 24 to March 10, 2018. Each subject recorded a “Symptom Diary” from one week before CCSNOP was installed to one week after panel removal. Since the “Cedar Project” was performed as a double-blind study, subjects did not know whether they were exposed to CCSNOP or the control panel. However, in this “Cypress Project,” all subjects were exposed to CCSNOP, since the number of subjects was limited. For the “Cedar Project,” subjects were examined for mood (using POMS2), stress (measured by salivary amylase), blood collections for blood counts, and general screening such as liver and kidney function, lipids, minerals, and immunoglobulins G, A, and M condition on January 13, February 24, March 11, and June 9, 2018. Additionally, 29 specific cytokines were measured using serum derived from the blood collections. For the “Cypress Project,” CCSNOP exposure was from April 1 to 15, 2018. Subjects recorded a “Symptom Diary” for 4 weeks as with the “Cedar Project.” Additionally, the same examinations were performed before and after CCSNOP exposure.

Ten Japanese volunteers with pollen allergies were part of this study. They were recruited for the experiment entitled “effects of cloth panel containing a specific ore powder on patients with Japanese cedar pollen allergy during the pollen dispersal season” (referred to as the # Cedar Project”) [22]. The results have already been published [22]. Subjects comprised two males (64 years old (y.o.) and 50 y.o.) and eight females. The average age was 41.4 ± 11.4 (standard deviation: SD) y.o. All 10 volunteers were living in the southern part of Okayama Prefecture and the sampling area comprised Kawasaki Medical School, Kurashiki, Japan, as shown in Figure 1(a). The living area of the volunteers is shown as a light blue oval. The map (Figure 1(a)) shows the locations where pollen volume measurements were performed, as previously published by Kimura [24].

As reported previously, these 10 volunteers were selected from a total of 31 volunteers recruited for the “Cedar Projects” [22]. Volunteers living in the Okayama area were selected for convenience of sampling. Pollen allergies against cypress as well as cedar were self-reported [22]. Volunteers typically mentioned experiencing pollen allergy symptoms for at least 5 years during the pollen dispersal period. All volunteers were free of other moderate to severe complications such as diabetes mellitus, hypertension, lung disease, cancer, or collagen disease. All subjects continued with their regular daily lives during the study without modification.

Since the number of volunteers was limited, all volunteers were exposed to CCSNOP. For this study, a negative (non-CCSNOP) control was not employed. The “Cedar Project” was performed as a double-blind test [22]. As mentioned in Introduction, all 10 subjects were surveyed for mood, stress, and the taking of blood on January 13 and June 9, 2018, as times representing the absence of pollen dispersal. Additionally, blood was sampled on February 24 and March 11, 2018, for the “Cedar Project.” Between these sampling dates, CCSNOP or control panels were present in volunteer bedrooms and all subjects kept a “Symptom Diary” from 1 week before panel installation until 1 week after panel removal.

For the “Cypress Project,” all 10 volunteers planned to keep a “Symptom Diary” from March 25 (1 week before panel installation) until April 21 (1 week after panel removal). Then, subjects were sampled for mood, stress, and the taking of blood on April 1 and April 15, 2018, at Kawasaki Medical School.

### 2.2. Ethical Matters

The “Cedar Project” was approved by the Kawasaki Medical School Ethics Committee (Issue No. 2,576, date of approval December 12, 2016). The “Cypress Project” was also approved for different issue by the Kawasaki Medical School Ethics Committee (Issue No. 2,982, date of approval February 19, 2018). All subjects of both projects approved verbally and in writing, and those from whom written consent was obtained were recruited as subjects in this study. Thus, 10 subjects were enlisted for the “Cypress Project” with each providing two forms of consent for each project. All methods used in these studies were performed in accordance with the relevant guidelines and regulations as outlined by the Kawasaki Medical School Ethics Committee as well as the Declaration of Helsinki.

### 2.3. Study Design

As shown in Figure 1(b), both “Cedar” and “Cypress” projects were performed from January to June, 2018. Samplings (POMS2, sAmy, and blood collection) were performed four times (No. a, 2, 3, and 6 in Figure 1(b)) [22] for the “Cedar Project” and two times (No. 4 and 5 in Figure 1(b)) for the “Cypress Project.” Numbers 1 and 6 sampling dates represented periods with no pollen dispersal. Numbers 2 and 3 represent sampling times for the “Cedar Project.” [22] Since cedar pollen dispersal begins in the middle of February and continues until near the end of March, 2018, use of CCSNOP or control panels encompassed the period from February 24 to March 11, 2018. All subjects kept a “Symptom Diary” [22] from one week before panel installation to one week after panel removal. As with the “Cedar Project,” all 10 subjects recruited for the “Cypress Project” employed the CCSNOP panel from April 1 until April 15, 2018. Furthermore, all subjects kept a “Symptom Diary” for 4 weeks that included the panel exposure period.

### 2.4. Cypress Pollen Dispersal in 2018

**Figure 2:**
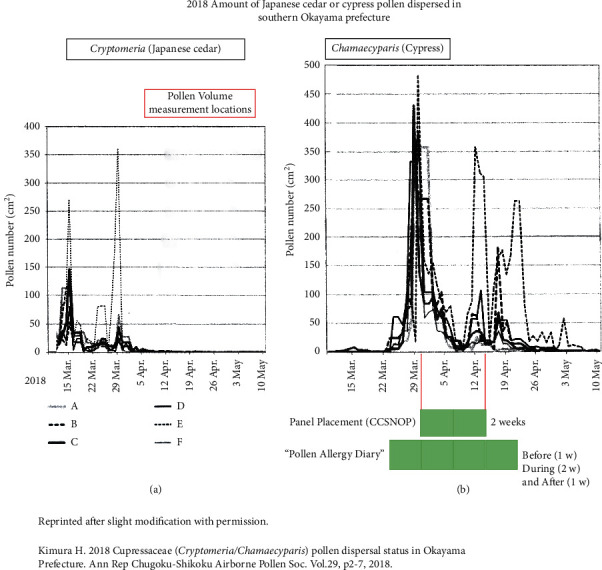
The amount of cypress pollen dispersed from the end of March to the end of April, 2018, in Okayama Prefecture, as obtained from a report by Kimura H (2018). The report is entitled “Cupressaceae (Cryptomeria/Chamaecyparis) pollen dispersal status in Okayama Prefecture.” Ann Rep Chugoku-Shikoku Airborne Pollen Soc. Vol. 29, p2-7, 2018, and is written in Japanese. Panels (a) and (b) show cedar and cypress pollen numbers, respectively, reprinted with permission by the author, Kimura, as well as the Editor-in-Chief, Dr. Fujiki. As shown in panel (a), cedar pollen dispersal ended at the end of March, 2018. On the other hand, as shown in panel (b), cypress pollen dispersal began in the middle of March and continued for more than one month. The lower part of panel (b) shows the periods encompassing CCSNOP panel exposure (2 weeks) and the keeping of a “Symptom Diary” (4 weeks). Although days with lower levels of pollen were observed (due to rain) during the 2-week period of CCSNOP exposure, the approximate average pollen dispersal seemed to be sufficient to evaluate the effects of CCSNOP. One week before panel installation also had sufficient pollen dispersal. However, one week after panel removal, pollen dispersal was relatively low. Only location E showed sufficient pollen dispersal during this period, although the majority of volunteers were not residing close to this location.

For the “Cedar Project,” periods of pollen dispersal were determined using pollen information presented by the Ministry of the Environment of Japan, called the “Hanako-san” system [22]. However, this system does not differentiate between cedar and cypress pollen. Therefore, a determination was required of the actual cypress pollen dispersal period in the Okayama area in 2018. A report entitled “Cupressaceae (*Cryptomeria/Chamaecyparis*) pollen dispersal status in Okayama Prefecture,” by Kimura, reported in Ann Rep Chugoku-Shikoku Airborne Pollen Soc. Vol. 29, pp. 2–7, 2018, was found [24]. With permission of the author, Kimura, as well as the Editor-in-Chief of this annual report, the findings of pollen dispersal of cedar and cypress in Okayama are reprinted here with slight modification and are shown in Figure 2(a). Sampling areas are shown in Figure 1(a). There were six locations (A to F) in the southern part of Okayama Prefecture. As shown in Figure 2(a), cedar pollen ceases to be dispersed from the end of March, 2018. However, cypress pollen (Figure 2(b)) dispersal begins in the middle of March and increases at the end of March. With less dispersal during a period comprising a few days around April 10 (informed as rainy), dispersal continues until the beginning of May, 2018. Thus, utilization of CCSNOP over a 2-week period appears adequate for our investigations. During these 2 weeks, cypress pollen was dispersed and we were able to compare data before (end of March) and during (first 2 weeks of April) panel use. However, after removal of CCSNOP, the amount of pollen dispersal decreased compared with that before panel use. This should be taken into account when the findings are evaluated.

### 2.5. Symptom Diary, Survey of Mood (POMS2), sAmy Measurement, and Blood Sampling

All these measurements were performed as described in our previous report for the “Cedar Project” [22].

### 2.6. Measurement of Cypress-Specific IgE and IgG4

The parameters or target molecules tested by blood sampling that differed from the “Cedar Project” include measurement of cypress-specific IgE and IgG4. These measurements were outsourced to Siemens Healthcare Diagnostics K.K., Tokyo, Japan. The sampled serum from all 10 subjects was shipped and measured by Siemens Healthcare Diagnostics K.K., and the results were delivered to researchers. Of course, these analyses were performed while maintaining the anonymity of the subjects involved in our studies.

### 2.7. Statistical Analyses

The statistical analyses were performed using SPSS version 22 (IBM, Chicago, IL, USA) or Microsoft Excel 2016 (Microsoft Japan, Tokyo, Japan). Changes in nose or eye symptoms during the three periods examined (before and during CCSNOP use and after CCSNOP removal) and the average number of days medication was utilized by subjects were initially checked by one-way ANOVA analysis. Thereafter, a test of significance was performed for the two groups using the Mann–Whitney *U* test. A chi-squared test was then performed using the cumulative number of days each subject utilized medication during the aforementioned three periods. Changes in the percentage or absolute number of eosinophils between blood collections before panel installation and after panel removal were analyzed by the Mann–Whitney *U* test. Changes in cypress-specific IgE and IgG4 among six blood collections were examined by one-way ANOVA analysis. Differences in serum concentration of individual cytokines between blood collections immediately before CCSNOP installation and just after panel removal were compared using the Mann–Whitney *U* test. For the statistical analyses, a *P* value of less than 0.05 was judged to be significant.

## 3. Results

### 3.1. Changes in Symptoms and Medication Use

**Figure 3:**
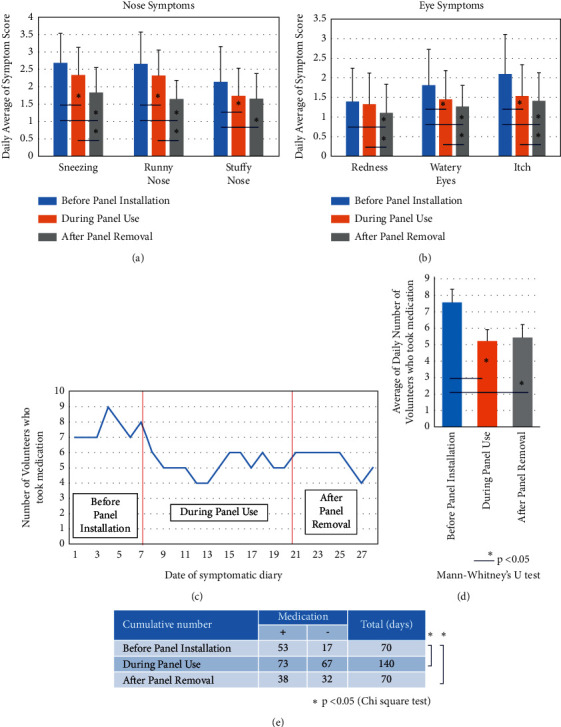
Analyses of symptoms from the “Symptom Diary.” (a) Comparison of nose symptoms such as sneezing, runny nose, and stuffy nose for the periods “before panel installation,” “during panel use,” and “after panel removal.” Scores were extracted from each “Symptom Diary” and the daily score average of the 10 subjects was plotted. (b) Comparison of eye symptoms such as redness, watery eyes, and itch during the periods “before panel installation,” “during panel use,” and “after panel removal.” Scores were extracted from each “Symptom Diary” and the daily score average of the 10 subjects was plotted. (c) Data pertaining to the use of medication as recorded in the “Symptom Diary” for each subject were plotted. (d) The average (with SD) daily number of volunteers who took medication in three periods “before panel installation,” “during panel use,” and “after panel removal” were plotted. In panels (a), (b), and (d), differences were initially examined by one-way ANOVA analysis, and then after confirmation of significance, a Mann–Whitney *U* test was performed to analyze the differences between the two groups. The ^*∗*^ indicates statistical significance, *P* < 0.05. (e) During the three periods “before panel installation,” “during panel use,” and “after panel removal,” the number of volunteers who took medication and the number of days were multiplied (medication + row), and the days and number of volunteers who did not take medication were also multiplied (medication–row). Differences between individual periods were then examined by a chi-squared test. The ^*∗*^ indicates significance, *P* < 0.05.

From the “Symptom Diary,” a symptom score (combining the daily score of 10 volunteers) was calculated. As shown in Figure 3(a), average scores (with SD) for nose symptoms such as sneezing, runny nose, and stuffy nose were determined. All of these symptoms diminished during the period immediately prior to and during use of CCSNOP. Similarly, symptom scores for all three symptoms decreased after the panel was removed. Furthermore, symptom scores for sneezing and runny nose following panel removal were lower compared to scores determined for the same symptoms during panel use.

Figure 3(b) shows average scores (with SD) for eye symptoms such as redness, watery eyes, and itch. Scores related to watery eyes and itch were lower before panel installation compared to during panel use. Similarly, scores after panel removal were lower for all three symptoms. Additionally, scores for all three symptoms were lower after panel removal compared to scores for the same symptoms during panel use. As mentioned above, it was noted that the period following panel removal comprised minimal pollen dispersal. Thus, the decreases observed in symptom scores might be due to the smaller amount of pollen dispersed. A comparison of scores obtained prior to panel installation with those obtained during panel use shows that five of the six symptoms examined had decreased.

Next, the use of medication was compared among the three periods examined (before and during CCSNOP use and after CCSNOP removal).  Figure 3(c) shows the average number of volunteers who took medication on each day during the 4-week period that diaries were kept. Numbers were higher before panel installation compared to the other two periods (during panel use and after panel removal). The average number of days when medication was utilized was determined and is shown in Figure 3(d). Again, the number was higher before panel installation compared to the other two periods. Moreover, a chi-squared test (Figure 3(e)) of the number of days medication was utilized showed a higher number before panel installation compared to the other two periods.

All of these findings indicated that exposure to CCSNOP while sleeping improved symptoms related to allergy against cypress pollen, and subjects decreased their intake of medication.

### 3.2. Mood and Stress Status

As with our previous reports [22], mood was examined using the POMS2 questionnaire, and stress status was determined by salivary amylase measurement. The results showed no significant differences (data not shown) before CCSNOP installation and after CCSNOP removal. Thus, CCSNOP did not affect the mood or stress status of volunteers with pollen allergy.

### 3.3. Changes in Eosinophils

**Figure 4:**
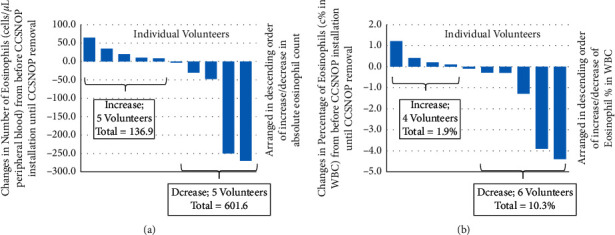
Comparison of the average change in the number (% multiplied by total WBC number/*μ*L) (a) or percentage of white blood cells (b) encompassing the period before installation of CCSNOP until removal of the CCSNOP panel for the 10 volunteers. The 5 left-most columns in (a) and 4 left-most columns in (b) increased and the total increase in number or percentage was added and indicated. 5 right-most columns in (a) and 6 right-most columns in (b) decreased. The total absolute number or percentage of increases or decreases is shown.

The percentage of eosinophils in white blood cells (WBCs) as well as the absolute number (number of WBCs multiplied by percentage divided by 100) were compared. Statistical analyses did not show any significant differences. However, as shown in Figure 4(a) for changes in number of eosinophils, the total increase among five volunteers was 136.9 cells/*μ*L, and the total decrease in number of eosinophils among five volunteers was 601.6 cells/*μ*L. Similarly, as shown in Figure 4(b) for changes in percentage of eosinophils in whole WBCs, the increase in percentage among four volunteers was 1.9%, while the total increase in percentage was 10.3%.

These results indicated that, in addition to allergy-related symptoms, CCSNOP affects eosinophils, which are the most important cells in patients with allergies in terms of standard blood sampling, as these cells play an important role in allergic inflammation. CCSNOP may reduce allergic inflammation by reducing eosinophil reaction.

### 3.4. Changes in Cypress-Specific IgE and IgG4

**Figure 5:**
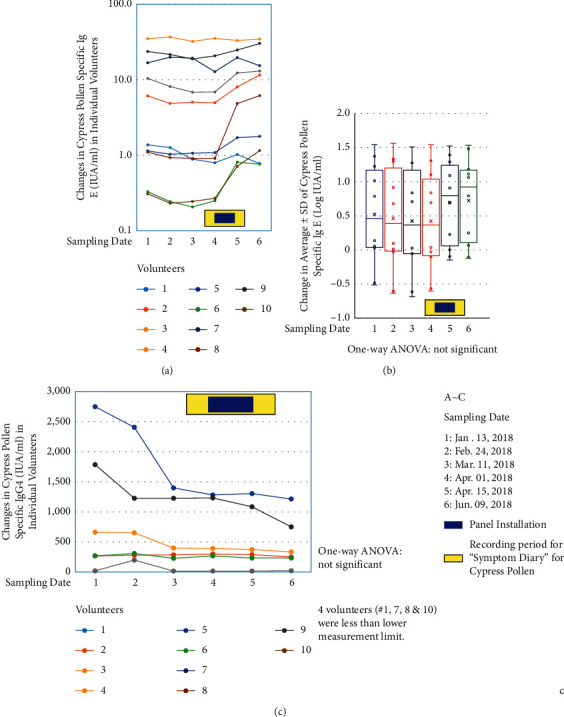
Changes in immunoglobulins. (a) Actual changes in cypress-specific IgE in 10 volunteers. The *X*-axis shows the six blood collection times described in Figure 2(b) and the lower right part of this figure. The *Y*-axis is represented logarithmically. As shown in the lower right part, the yellow box shows the period when the “Symptom Diary” was kept for cypress pollen allergy, while the blue box indicates the 2 weeks of exposure to CCSNOP. (b) The data in panel A are shown as box plots of cypress-specific IgE of 10 volunteers with average ± standard deviations. The *Y*-axis is shown as Log IgE IUA/ml. There were no significant differences identified among these data by one-way ANOVA analysis. The blue and yellow boxes are the same as in panel (a). Sampling dates # 1 to 6 are the same as in Figure 2(b) and are shown in the lower right part of this figure. (c) Changes in cypress-specific IgG4 (IUA/ML) in individual volunteers, except volunteers # 1, 7, 8, and 10 whose numbers are written in gray. These four volunteers showed less than the measurement lower limit (200 IUA/ml) for all six sampling times. There were no statistical differences between sampling times as determined by one-way ANOVA analysis. The blue and yellow boxes are the same as in panel (a). Sampling dates # 1 to 6 are the same as in Figure 2(b) and are shown in the lower right part of this figure.

All volunteers had blood taken four times in the “Cedar Projects” and an additional two times in the “Cypress Project.” As shown in Figure 5(a), changes in cypress-specific IgE for each volunteer are plotted for the six blood collection times. The average of six time points is shown as box plots (Figure 5(b)). There was no significant difference among the six time points regarding the value of cypress-specific IgE. Although statistically there were no significant changes, as shown in Figure 5(a), levels of cypress-specific IgE tended to increase in 9 out of 10 volunteers. It seems that, from a physiological standpoint, levels of cypress-specific IgE tended to increase in the body of subjects with pollen allergies. However, even with this situation, use of CCSNOP resulted in reduced allergy-related symptoms.

Figure 5(c) shows the changes in cypress-specific IgG4. Four volunteers (# 1, 7, 8, and 10) showed values less than the measurement lower limit for all six measurements. Volunteer # 3 had only one value greater than the measurement lower limit, with the remaining five values being less than the measurement lower limit. Although these changes were not statistically significant, it is noteworthy that three volunteers showed relatively higher cypress-specific IgG4 values at blood collection time points 1 and 2. However, these values decreased after time points 3 or 4, when cedar as well as cypress pollen began to be dispersed. If cypress-specific IgG4 is considered as a decoy antibody against pollen or an inhibitory antibody against cypress-specific IgE [25–27], some pollen allergy patients may possess relatively higher levels of cypress-specific IgG4 after long-term exposure to pollens. However, at the period when pollen dispersal was marked, IgG4 could not effectively inhibit IgE as patients have been exposed to many pollens. These amounts of pollen did not seem to be countered by the small amount of cypress-specific IgG4. Thus, some subjects showed slightly higher levels of cypress-specific IgG4 during the winter time, before pollen dispersal, and after dispersal levels of IgG4 decreased.

### 3.5. Changes in Cytokines

**Figure 6:**
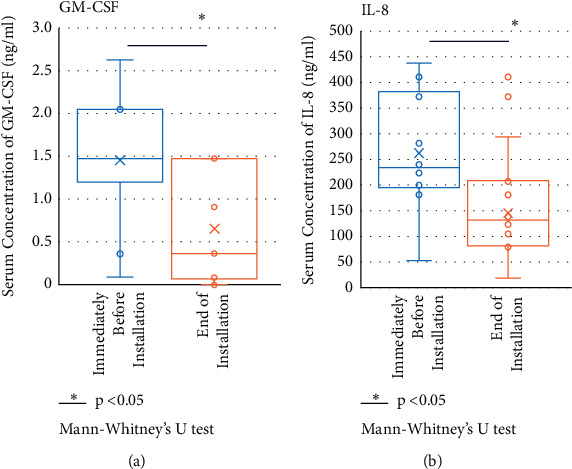
Examination of 29 cytokines from blood collections just before CCSNOP exposure and immediately before removal of CCSNOP panel (blood collections # 4 and 5, in Figure 2). GM-CSF (panel (a)) and IL8 (panel (b)) showed significant differences as determined by the Mann–Whitney *U* test (*P* < 0.05). Values for both cytokines were higher just before CCSNOP installation compared to those immediately prior to CCSNOP removal.

Similar to our previous report detailing the results of the “Cedar Project” [22], in this study (“Cypress Project”) 29 kinds of serum cytokines were measured and changes in these cytokines were determined before installation of CCSNOP and after CCSNOP removal. Results showed that significant changes were only observed for cytokines GM-CSF and IL8 (Figures 6(a) and 6(b), respectively), as determined by the Mann–Whitney *U* test. Levels of both cytokines decreased after CCSNOP removal. These results indicated that use of CCSNOP may affect the immune system in the body or that CCSNOP only reduces allergy-related symptoms. However, even when for 2 weeks symptoms had decreased, pollen dispersal altered the immune system. Thus, CCSNOP may indirectly affect the human immune system. GM-CSF is extracted and decreasing GM-CSF indicates individuals exposed to CCSNOP and not the control panel. This is suitable for use in the “Cypress Project.” GM-CSF was significantly reduced, and this cytokine may possess some function related to allergy and inflammation. The observed decrease in GM-CSF and reduction of symptoms were adequate and matched. Additionally, levels of cytokine IL8, a typical inflammatory cytokine, were reduced. It seems that use of CCSNOP may modify and reduce allergy-related inflammation, in addition to reducing allergy symptoms. CCSNOP might act in a direct manner to decrease levels of important cytokines. Thus, the immune effects of CCSNOP may act in a direct as well as in an indirect manner.

## 4. Discussion

The diminution of pollen allergy involves many aspects [1–3], with efforts to reduce pollen dispersal perhaps being the most important [28–30]. After World War II, the Japanese Ministry decided to plant many cedar and cypress trees in an effort to enhance forest regions [31]. Japanese cedar and cypress trees are therefore present in many mountainous regions of Japan [32]. Consequently, this has led to marked dispersal of cedar and cypress pollen at the end of the previous century. Since it is difficult to immediately reduce pollen dispersal, symptomatic therapy using antihistamines represents one effective strategy to counter pollen-related allergies. Cases of pollen allergy are addressed by symptomatic treatment rather than curative treatment. This is because they do not have to be particularly aware of the disease at the end of the pollen season, although the symptoms also have an impact on the daily lives of affected individuals. Of course, allergen immunotherapy can be performed as one method involving radical treatment [33–35]. However, this approach is not widespread since pollen allergy is seasonal and is generally not a life-threatening disease. Another strategy usually employed is the confinement of allergy sufferers to indoor environments such as homes, offices, and other places. Preventing or limiting pollen entry into buildings and homes may also be effective. On the other hand, there is the possibility that pollen can be transported into indoor areas via attachment to clothes and hair.

Our device, referred to as CCSNOP, was developed as a result of customer feedback and anecdotal reports [21, 22]. Clients of Cosmic Garden Co. Ltd. live in detached dwellings. All of these houses include CCSNOP as part of the interior material inside the walls. Clients experienced a reduction of pollen-related symptoms in addition to improved sleeping and a better state of mind. Thus, with collaboration between Cosmic Garden Co. Ltd., Okayama, Japan, and the Department of Hygiene, Kawasaki Medical School, Kurashiki, Japan, the effects of CCSNOP on pollen allergy have been investigated [21, 22].

At the first, the one-hour stay experiment was performed [21]. Subjects with pollen allergies (at this time, cedar or cypress was not defined, just self-reported) stayed for one hour in a room surrounded by control cloth. Then, before and after the stay, a symptom questionnaire, POMS2, sAmy, and blood collection were performed. One week later, subjects stayed again for one hour in the same room; only this time the cloth was CCSNOP. The questionnaire, POMS2, sAmy, and blood collection were the same. It was found that some symptoms such as stuffy nose and watery eyes improved [21]. Thereafter, POMS was stable. However, levels of eosinophils, nonspecific IgE, epidermal growth factor (EGF), monocyte chemotactic protein- (MCP-) 1, and tumor necrosis factor- (TNF-) *α* increased following exposure to CCSNOP. The reduction of symptoms matched expectations [21]. However, other results were less comprehensible. Then, during pollen season, it was considered necessary to have volunteers with pollen allergies stay in their home for at least 2 weeks under observation. Then, the “Cedar Project” was developed [22].

As reported previously, the “Cedar Project” was performed as a double-blind test and the results are highly reliable. Use of CCSNOP relieved symptoms and led to a reduction in use of therapeutics. Moreover, subjects with higher eosinophil counts before installation of CCSNOP showed a marked decrease in eosinophils. Finally, the “panel-identifying” formula (mentioned above) was formed. This formula indicated that CCSNOP may affect the immune system of pollen allergy subjects [22].

During execution of the “Cedar Project,” we had the opportunity to collaborate with Siemens Healthcare Diagnostics K.K. regarding the measurement of cypress-specific IgE as well as IgG4. CCSNOP installation was performed from the end of February to early March, 2018. This period is considered to encompass the dispersal of cedar pollen, although cypress pollen dispersal encompasses the end of March to the end of April. Thus, 10 subjects were recruited from 31 pollen allergy patients in the “Cedar Project.” Although the “Cedar Project” was performed as a double-blind experiment, the “Cypress Project” was somewhat limited due to the smaller number (10) of subjects involved. Thus, for the latter experiments, all subjects were exposed to CCSNOP in their bedrooms. Thereafter, collation of data from a “Symptom Diary,” POMS2, sAmy, and blood collections were performed as in previous investigations [21, 22].

Results showed that exposure to CCSNOP reduced symptoms and that subjects decreased their intake of medication. It is clear that CCSNOP improves symptoms of pollen allergy and reduces the requirement for treatment. It then became necessary to determine the mechanism or manner by which CCSNOP affects the human body. The effect of CCSNOP on changes in cypress-specific IgE appeared to be negligible. Levels of cypress-specific IgE tended to increase in certain subjects (although not statistically significant). Moreover, in certain subjects levels of cypress-specific IgG4 [25, 27, 36–38], the decoy or inhibitor of cypress-specific IgE against cypress pollen, decreased after the beginning of pollen dispersal. This represents a reversal of the changes observed for cypress-specific IgE. These findings do not indicate that CCSNOP acts in a direct manner to affect the human immune system but may act by reducing the amount of pollen in a room, thereby leading to a reduction in allergy symptoms. However, if subjects were exposed to less pollen, then the observed changes in cypress-specific IgE and IgG4 should not have been as marked. Thus, hitherto unknown mechanisms related to the use of CCSNOP may be at play to reduce allergy symptoms.

Although we had investigated the effects of CCSNOP mainly on clinical observations such as symptom, dosage of medication, and other parameters, to explore the mechanistic effects of CCSNOP is very difficult. These might be nonspecific or specific and dependent on the CCSNOP immunomodulatory properties. Although volcano-derived ore may contain some immunotoxic compounds just as fumes released during volcano eruption, our previous investigation of mineralogical aspects indicated no special features in this ore. The CCSNOP only emitted far infrared rays lightly. It would be beneficial to make some basic in vitro experiments to test immunological activity of ore powder to ensure safety of using CCSNOP. However, Cosmic Garden Co. Ltd. have been selling detached house more than 20 years with CCSNOP. There are no issues regarding health problem among house-owners. Thus, these facts indicated CCSNOP is basically safe for humans. In addition, it may possess immunomodulating activity.

Additionally, the decreased levels of cytokines GM-SCF and IL8 seemed to indicate that CCSNOP may have a direct effect on the human immune system. In particular, CCSNOP might act to reduce allergic inflammation, since both cytokines act as enhancers of allergic inflammation. Ito et al. reported the establishment of a human allergy model using human IL-3/GM-CSF-transgenic NOG mice [39]. They showed that a large number of human myeloid cells of various lineages developed after transplantation of human CD34^−^ hematopoietic stem cells. Notably, the number of mature basophils and mast cells expressing a high-affinity IgE receptor (also known as Fc*ε*RI or Fc epsilon RI) was markedly increased. Thus, our findings showing a decrease in GM-CSF in subjects before CCSNOP installation up until after panel removal may indicate the occurrence of a molecular reduction in allergic reactions. Moreover, although these were relatively old investigations, Linden et al. reported that GM-CSF may play a role *in vivo* to increase the production of eosinophilic progenitors in allergic airway disease [40, 41]. Their study examined mature eosinophils (nasal and circulating), their circulating progenitors, and the potential role of GM-CSF in stimulating these progenitors. Recruiting 12 subjects with pollen allergy during pollen season, all subjects showed severe symptoms and an increase in eosinophils in nasal mucosa and peripheral blood. Additionally, levels of GM-CSF in nasal lavage fluids increased. Their *in vitro* experiment showed that GM-CSF could respond to progenitor cells of basophils and eosinophils [40, 41].

Interleukin 8 (IL8) is a well-known typical inflammatory cytokine. Gokkaya et al. recently reported that nasal pollen-specific IgA and IgG isotypes are potentially protective within the humoral compartment [42]. Additionally, they concluded that nasal levels of IL8, IL33, certain pollen-specific IgG4, and certain pollen-specific IgE could be predictive biomarkers for pollen-specific symptom expression [43]. Gaspar et al. also reported recently that pollen extracts induced an increase in the release of IL6 and IL8 cytokines, as measured by flow cytometry, possibly as a result of the activation of protease-activated receptor 2 (PAR-2), based on the fact that pollens are important triggers for allergic rhinitis, conjunctivitis, and asthma [43]. Proteases released upon pollen grain hydration appear to play a major role in the typical immunological and inflammatory responses that occur in people with allergic disorders [43].

Taken together, the reduction in GM-CSF and IL8 observed during use of CCSNOP may indicate that CCSNOP has a direct effect on the human immune system and that these effects may result in reduced symptoms in people with pollen allergies. However, the precise manner by which CCSNOP affects the human body to reduce pollen allergy symptoms remains to be determined and is especially challenging as the natural ore, which comprises the powdered material of CCSNOP, is not a unique or specific ore.

On the other viewpoint, panel may decrease concentration of all allergens present in rooms resulting in GM-CSF and IL-8 decrease in patients. For example, house dust mite (HDM) allergens induce the release of cytokines eotaxin, GM-CSF, CCL2, CCL20, IL-6, and IL-8; cockroach allergens have been shown to induce IL-6, IL-8, and GM-CSF and fungal allergens such as *Alternaria* have been implicated in the release of IL-6, GM-CSF, and IL-33 from airway epithelial cells [44]. People can also imagine a nonspecific action of CCSNOP. Probably due to porous structure of the panels they may act as a sponge and adsorb pollens and other allergens on its surface. However, at our initial experiment, we were not able to show these changes [21]. Future studies will need to elucidate pollen dynamics and the role of CCSNOP.

Future investigations comprising a long-term prospective study will need to enlist a large number of customers who are living in CCSNOP homes. Since it might be difficult to develop a satisfactory animal model suitable for CCSNOP investigations, one strategy might be to breed allergic animals in CCSNOP cages, which could then be employed to identify molecular targets using various omics studies.

## 5. Conclusion

The effects of CCSNOP were investigated with respect to pollen allergy symptoms, mood, stress status, and changes in peripheral blood (especially eosinophils and cytokines) by blood sampling. Results showed that use of CCSNOP led to reduced nasal and eye symptoms and that participating subjects decreased their intake of medication. Although CCSNOP had no effect on mood or stress status, eosinophil numbers were reduced. More importantly, exposure to CCSNOP caused a decrease in the levels of allergic inflammatory cytokines such as GM-SCF and IL8. Thus, CCSNOP may be useful in reducing symptoms related to cypress pollen allergy, as well as cedar allergy as previously reported. Long-term observation of residents in CCSNOP environments will be needed in an effort to evaluate chronic effects.

## Data Availability

The measured and analyzed data used to support the findings of this study are included within the article.
